# A practical guide to amplicon and metagenomic analysis of microbiome data

**DOI:** 10.1007/s13238-020-00724-8

**Published:** 2020-05-11

**Authors:** Yong-Xin Liu, Yuan Qin, Tong Chen, Meiping Lu, Xubo Qian, Xiaoxuan Guo, Yang Bai

**Affiliations:** 1 State Key Laboratory of Plant Genomics, Institute of Genetics and Developmental Biology, Chinese Academy of Sciences, Beijing, 100101 China; 2 CAS Center for Excellence in Biotic Interactions, University of Chinese Academy of Sciences, Beijing, 100049 China; 3 CAS-JIC Centre of Excellence for Plant and Microbial Science, Institute of Genetics and Developmental Biology, Chinese Academy of Sciences, Beijing, 100101 China; 4 College of Advanced Agricultural Sciences, University of Chinese Academy of Sciences, Beijing, 100049 China; 5 National Resource Center for Chinese Materia Medica, China Academy of Chinese Medical Sciences, Beijing, 100700 China; 6 Department of Rheumatology Immunology & Allergy, Children’s Hospital, Zhejiang University School of Medicine, Hangzhou, Zhejiang Province 310053 China

**Keywords:** metagenome, marker genes, high-throughput sequencing, pipeline, reproducible analysis, visualization

## Abstract

Advances in high-throughput sequencing (HTS) have fostered rapid developments in the field of microbiome research, and massive microbiome datasets are now being generated. However, the diversity of software tools and the complexity of analysis pipelines make it difficult to access this field. Here, we systematically summarize the advantages and limitations of microbiome methods. Then, we recommend specific pipelines for amplicon and metagenomic analyses, and describe commonly-used software and databases, to help researchers select the appropriate tools. Furthermore, we introduce statistical and visualization methods suitable for microbiome analysis, including alpha- and beta-diversity, taxonomic composition, difference comparisons, correlation, networks, machine learning, evolution, source tracing, and common visualization styles to help researchers make informed choices. Finally, a step-by-step reproducible analysis guide is introduced. We hope this review will allow researchers to carry out data analysis more effectively and to quickly select the appropriate tools in order to efficiently mine the biological significance behind the data.

## Introduction

Microbiome refers to an entire microhabitat, including its microorganisms, their genomes, and the surrounding environment (Marchesi and Ravel, 2015). With the development of high-throughput sequencing (HTS) technology and data analysis methods, the roles of the microbiome in humans (Gao et al., 2018; Yang and Yu, 2018; Zhang et al., 2018a), animals (Liu et al., 2020), plants (Liu et al., 2019a; Wang et al., 2020a), and the environment (Mahnert et al., 2019; Zheng et al., 2019) have gradually become clearer in recent years. These findings have completely changed our understanding of the microbiome. Several countries have launched successful international microbiome projects, such as the NIH Human Microbiome Project (HMP) (Turnbaugh et al., 2007), the Metagenomics of the Human Intestinal Tract (MetaHIT) (Li et al., 2014), the integrative HMP (iHMP) (Proctor et al., 2019), and the Chinese Academy of Sciences Initiative of Microbiome (CAS-CMI) (Shi et al., 2019b). These projects have made remarkable achievements, which have pushed microbiome research into a golden era.

The framework for amplicon and metagenomic analysis was established in the last decade (Caporaso et al., 2010; Qin et al., 2010). However, microbiome analysis methods and standards have been evolving rapidly over the past few years (Knight et al., 2018). For example, there was a proposal to replace operational taxonomic units (OTUs) with amplicon sequence variants (ASVs) in marker gene-based amplicon data analysis (Callahan et al., 2016). The next-generation microbiome analysis pipeline QIIME 2, a reproducible, interactive, efficient, community-supported platform was recently published (Bolyen et al., 2019). In addition, new methods have recently been proposed for taxonomic classification (Ye et al., 2019), machine learning (Galkin et al., 2018), and multi-omics integrated analysis (Pedersen et al., 2018).

The development of HTS and analysis methods has provided new insights into the structures and functions of microbiome (Jiang et al., 2019; Ning and Tong, 2019). However, these new developments have made it challenging for researchers, especially those without a bioinformatics background, to choose suitable software and pipelines. In this review, we discuss the widely used software packages for microbiome analyses, summarize their advantages and limitations, and provide sample codes and suggestions for selecting and using these tools.

## HTS methods of microbiome analysis

**Figure 1:**
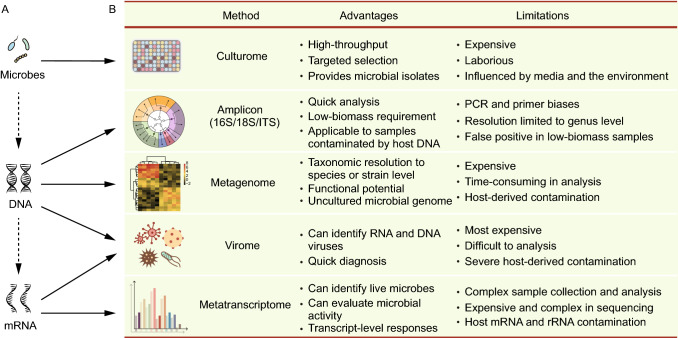
**Advantages and limitations of HTS methods used in microbiome research**. A Introduction to HTS methods for different levels of analysis. At the molecule-level, microbiome studies are divided into three types: microbe, DNA, and mRNA. The corresponding research techniques include culturome, amplicon, metagenome, metavirome, and metatranscriptome analyses. B The advantages and limitations of various HTS methods for microbiome analysis

The first step in microbiome research is to understand the advantages and limitations of specific HTS methods. These methods are primarily used for three types of analysis: microbe-, DNA-, and mRNA-level analyses (Fig. 1A). The appropriate method(s) should be selected based on sample types and research goals.

Culturome is a high-throughput method for culturing and identifying microbes at the microbe-level (Fig. 1A). The microbial isolates are obtained as follows. First, the samples are crushed, empirically diluted in liquid medium, and distributed in 96-well microtiter plates or Petri dishes. Second, the plates are cultured for 20 days at room temperature. Third, the microbes in each well are subjected to amplicon sequencing, and wells with pure, non-redundant colonies are selected as candidates. Fourth, the candidates are purified and subjected to 16S rDNA full-length Sanger sequencing. Finally, the newly characterized pure isolates are preserved (Zhang et al., 2019). Culturome is the most effective method for obtaining bacterial stocks, but it is expensive and labor intensive (Fig. 1B). This method has been used for microbiome analysis in humans (Goodman et al., 2011; Zou et al., 2019), mouse (Liu et al., 2020), marine sediment (Mu et al., 2018), *Arabidopsis thaliana* (Bai et al., 2015), and rice (Zhang et al., 2019). These studies not only expanded the catalog of taxonomic and functional databases for metagenomic analyses, but also provided bacterial stocks for experimental verification. For further information, please see (Lagier et al., 2018; Liu et al., 2019a).

DNA is easy to extract, preserve, and sequence, which has allowed researchers to develop various HTS methods (Fig. 1A). The commonly used HTS methods of microbiome are amplicon and metagenomic sequencing (Fig. 1B). Amplicon sequencing, the most widely used HTS method for microbiome analysis, can be applied to almost all sample types. The major marker genes used in amplicon sequencing include 16S ribosome DNA (rDNA) for prokaryotes and 18S rDNA and internal transcribed spacers (ITS) for eukaryotes. 16S rDNA amplicon sequencing is the most commonly used method, but there is currently a confusing array of available primers. A good method for selecting primer is to evaluate their specificity and overall coverage using real samples or electronic PCR based on the SILVA database (Klindworth et al., 2012) and on host factors including the presence of chloroplasts, mitochondria, ribosomes, and other potential sources of non-specific amplification. Alternatively, researchers can refer to the primers used in published studies similar to their own, which would save time in method optimization and facilitate to compare results among studies. Two-step PCR is typically used for amplification and to add barcodes and adaptors to each sample during library preparation (de Muinck et al., 2017). Sample sequencing is often performed on the Illumina MiSeq, HiSeq 2500, or NovaSeq 6000 platform in paired-end 250 bases (PE250) mode, which generates 50,000–100,000 reads per sample. Amplicon sequencing can be applied to low-biomass specimens or samples contaminated by host DNA. However, this technique can only reach genus-level resolution. Moreover, it is sensitive to the specific primers and number of PCR cycles chosen, which may lead to some false-positive or false-negative results in downstream analyses (Fig. 1B).

Metagenomic sequencing provides more information than amplicon sequencing, but it is more expensive using this technique. For ‘pure’ samples such as human feces, the accepted amount of sequencing data for each sample ranges from 6 to 9 gigabytes (GB) in a metagenomic project. The corresponding price for library construction and sequencing ranges from $100 to $300. For samples containing complex microbiota or contaminated with host-derived DNA, the required sequencing output ranges from 30 to 300 GB per sample (Xu et al., 2018). In brief, 16S rDNA amplicon sequencing could be used to study bacteria and/or archaea composition. Metagenomic sequencing is advisable for further analysis if higher taxonomic resolution and functional information are required (Arumugam et al., 2011; Smits et al., 2017). Of course, metagenomic sequencing could be used directly in studies with smaller sample sizes, assuming sufficient project funding is available (Carrión et al., 2019; Fresia et al., 2019).

Metatranscriptomic sequencing can profile mRNAs in a microbial community, quantify gene expression levels, and provide a snapshot for functional exploration of a microbial community *in situ* (Turner et al., 2013; Salazar et al., 2019). It is worth noting that host RNA and other rRNAs should be removed in order to obtain transcriptional information of microbiota (Fig. 1B).

Since viruses have either DNA or RNA as their genetic materials, technically, metavirome research involves a combination of metagenome and metatranscriptome analyses (Fig. 1A and 1B). Due to the low biomass of viruses in a sample, virus enrichment (Metsky et al., 2019) or the removal of host DNA (Charalampous et al., 2019) is essential steps for obtaining sufficient quantities of viral DNA or RNA for analysis (Fig. 1B).

The selection of sequencing methods depends on the scientific questions and sample types. The integration of different methods is advisable, as multi-omics provides insights into both the taxonomy and function of the microbiome. In practice, most researchers select only one or two HTS methods for analysis due to time and cost limitations. Although amplicon sequencing can provide only the taxonomic composition of microbiota, it is cost effective ($20–50 per sample) and can be applied to large-scale research. In addition, the amount of data generated from amplicon sequencing is relatively small, and the analysis is quick and easy to perform. For example, data analysis of 100 amplicon samples could be completed within a day using an ordinary laptop computer. Thus, amplicon sequencing is often used in pioneering research. In contrast to amplicon sequencing, metagenomic sequencing not only extends taxonomic resolution to the species- or strain-level but also provides potential functional information. Metagenomic sequencing also makes it possible to assemble microbial genomes from short reads. However, it does not perform well for low-biomass samples or those severely contaminated by the host genome (Fig. 1B).

## Analysis pipelines

“Analysis pipeline” refers to a particular program or script that combines several or even dozens of software programs organically in a certain order to complete a complex analysis task. As of January 23, 2020, the words “amplicon” and “metagenome” were mentioned more than 200,000 and 40,000 times in Google Scholar, respectively. Due to their wide usage, we will discuss the current best-practice pipelines for amplicon and metagenomic analysis. Researchers should get acquainted with the Shell environment and R language, which we discussed in our previous review (Liu et al., 2019b).

### Amplicon analysis

**Figure 2:**
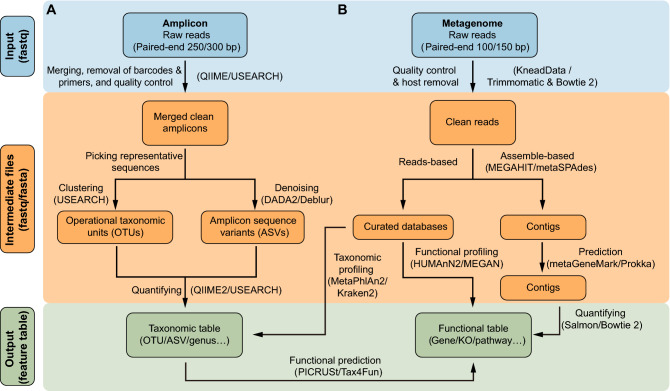
**Workflow of commonly used methods for amplicon (A) and metagenomic (B) sequencing.** Blue, orange, and green blocks represent input, intermediate, and output files, respectively. The text next to the arrow represents the method, with frequently used software shown in parentheses. Taxonomic and functional tables are collectively referred to as feature tables. Please see Table 1 for more information about the software listed in this figure

The first stage of amplicon analysis is to convert raw reads (typically in fastq format) into a feature table (Fig. 2A). The raw reads are usually in paired-end 250 bases (PE250) mode and generated from the Illumina platforms. Other platforms, including Ion Torrent, PacBio, and Nanopore, are not discussed in this review and may not be suitable for the analysis pipelines discussed below. First, raw amplicon paired-end reads are grouped based on their barcode sequences (demultiplexing). Then the paired reads are merged to obtain amplicon sequences, and barcode and primers are removed. A quality-control step is normally needed to remove low-quality amplicon sequences. All of these steps can be completed using USEARCH (Edgar, 2010) or QIIME (Caporaso et al., 2010). Alternatively, clean amplicon data supplied by sequencing service providers can be used for next analysis (Fig. 2A).

Picking the representative sequences as proxies of a species is a key step in amplicon analysis. Two major approaches for representative sequence selection are clustering to OTUs and denoising to ASVs. The UPARSE algorithm clusters sequences with 97% similarity into OTUs (Edgar, 2013). However, this method may fail to detect subtle differences among species or strains. DADA2 is a recently developed denoising algorithm that outputs ASVs as more exactly representative sequences (Callahan et al., 2016). The denoising method is available at *denoise-paired*/*single* by DADA2, *denoise-16S* by Deblur in QIIME 2 (Bolyen et al., 2019), and *-unoise3* in USEARCH (Edgar and Flyvbjerg, 2015). Finally, a feature table (OTU/ASV table) can be obtained by quantifying the frequency of the feature sequences in each sample. Simultaneously, the feature sequences can be assigned taxonomy, typically at the kingdom, phylum, class, order, family, genus, and species levels, providing a dimensionality reduction perspective on the microbiota.

In general, 16S rDNA amplicon sequencing can only be used to obtain information about taxonomic composition. However, many available software packages have been developed to predict potential functional information. The principle behind this prediction is to link the 16S rDNA sequences or taxonomy information with functional descriptions in literature. PICRUSt (Langille et al., 2013), which is based on the OTU table of the Greengenes database (McDonald et al., 2011), could be used to predict the metagenomic functional composition (Zheng et al., 2019) of Kyoto Encyclopedia of Genes and Genomes (KEGG) pathways (Kanehisa and Goto, 2000). The newly developed PICRUSt2 software package (https://github.com/picrust/picrust2) can directly predict metagenomic functions based on an arbitrary OTU/ASV table. The R package Tax4Fun (Asshauer et al., 2015) can predict KEGG functional capabilities of microbiota based on the SILVA database (Quast et al., 2013). The functional annotation of prokaryotic taxa (FAPROTAX) pipeline performs functional annotation based on published metabolic and ecological functions such as nitrate respiration, iron respiration, plant pathogen, and animal parasites or symbionts, making it useful for environmental (Louca et al., 2016), agricultural (Zhang et al., 2019), and animal (Ross et al., 2018) microbiome research. BugBase is an extended database of Greengenes used to predict phenotypes such as oxygen tolerance, Gram staining, and pathogenic potential (Ward et al., 2017); this database is mainly used in medical research (Mahnert et al., 2019).

### Metagenomic analysis

Compared to amplicon, shotgun metagenome can provide functional gene profiles directly and reach a much higher resolution of taxonomic annotation. However, due to the large amount of data, the fact that most software is only available for Linux systems, and the large amount of computing resources are needed to perform analysis. To facilitate software installation and maintenance, we recommend using the package manager Conda with BioConda channel (Grüning et al., 2018) to deploy metagenomic analysis pipelines. Since metagenomic analysis is computationally intensive, it is better to run multiple tasks/samples in parallel, which requires software such as GNU Parallel for queue management (Tange, 2018).

The Illumina HiSeqX/NovaSeq system often produces PE150 reads for metagenomic sequencing, whereas reads generated by BGI-Seq500 are in PE100 mode. The first crucial step in metagenomic analysis is quality control and the removal of host contamination from raw reads, which requires the KneadData pipeline (https://bitbucket.org/biobakery/kneaddata) or a combination of Trimmomatic (Bolger et al., 2014) and Bowtie 2 (Langmead and Salzberg, 2012). Trimmomatic is a flexible quality-control software package for Illumina sequencing data that can be used to trim low-quality sequences, library primers and adapters. Reads mapped to host genomes using Bowtie 2 are treated as contaminated reads and filtered out. KneadData is an integrated pipeline, including Trimmomatic, Bowtie 2, and related scripts that can be used for quality control, to remove host-derived reads, and to output clean reads (Fig. 2B).

The main step in metagenomic analysis is to convert clean data into taxonomic and functional tables using reads-based and/or assembly-based methods. The reads-based methods align clean reads to curated databases and output feature tables (Fig. 2B). MetaPhlAn2 is a commonly used taxonomic profiling tool that aligns metagenome reads to a pre-defined marker-gene database to perform taxonomic classification (Truong et al., 2015). Kraken 2 performs exact *k*-mer matching to sequences within the NCBI non-redundant database and uses lowest common ancestor (LCA) algorithms to perform taxonomic classification (Wood et al., 2019). For a review about benchmarking 20 tools of taxonomic classification, please see Ye et al. (2019). HUMAnN2 (Franzosa et al., 2018), the widely used functional profiling software, can also be used to explore within- and between-sample contributional diversity (species’ contributions to a specific function). MEGAN (Huson et al., 2016) is a cross-platform graphical user interface (GUI) software that performs taxonomic and functional analyses (Table 1). In addition, various metagenomic gene catalogs are available, including catalogs curated from the human gut (Li et al., 2014; Pasolli et al., 2019; Tierney et al., 2019), the mouse gut (Xiao et al., 2015), the chicken gut (Huang et al., 2018), the cow rumen (Stewart et al., 2018; Stewart et al., 2019), the ocean (Salazar et al., 2019), and the citrus rhizosphere (Xu et al., 2018). These customized databases can be used for taxonomic and functional annotation in the appropriate field of study, allowing efficient, precise, rapid analysis.

**Table 1:** Introduction to software for amplicon and metagenomic analysis

Name	Link	Description and advantages	Reference
QIIME	http://qiime.org	The most highly cited and comprehensive amplicon analysis pipeline, providing hundreds of scripts for analyzing various data types and visualizations	(Caporaso et al., 2010)
QIIME 2	https://qiime2.org https://github.com/YongxinLiu/QIIME2ChineseManual	This next-generation amplicon pipeline provides integrated command lines and GUI, and supports reproducible analysis and big data. Provides interactive visualization and Chinese tutorial documents and videos	(Bolyen et al., 2019)
USEARCH	http://www.drive5.com/usearch https://github.com/YongxinLiu/UsearchChineseManual	Alignment tool includes more than 200 subcommands for amplicon analysis with a small size (1 Mb), cross-platform, high-speed calculation, and free 32-bit version. The 64-bit version is commercial ($1485)	(Edgar, 2010)
VSEARCH	https://github.com/torognes/vsearch	A free USEARCH-like software tool. We recommend using it alone or in addition to USEARCH. Available as a plugin in QIIME 2	(Rognes et al., 2016)
Trimmomatic	http://www.usadellab.org/cms/index.php?page=trimmomatic	Java based software for quality control of metagenomic raw reads	(Bolger et al., 2014)
Bowtie 2	http://bowtie-bio.sourceforge.net/bowtie2	Rapid alignment tool used to remove host contamination or for quantification	(Langmead and Salzberg, 2012)
MetaPhlAn2	https://bitbucket.org/biobakery/metaphlan2	Taxonomic profiling tool with a marker gene database from more than 10,000 species. The output is relative abundance of strains	(Truong et al., 2015)
Kraken 2	https://ccb.jhu.edu/software/kraken2	A taxonomic classification tool that uses exact *k*-mer matches to the NCBI database, high accuracy and rapid classification, and outputs reads counts for each species	(Wood et al., 2019)
HUMAnN2	https://bitbucket.org/biobakery/humann2	Based on the UniRef protein database, calculates gene family abundance, pathway coverage, and pathway abundance from metagenomic or metatranscriptomic data. Provide species’ contributions to a specific function	(Franzosa et al., 2018)
MEGAN	https://github.com/husonlab/megan-ce http://www-ab.informatik.uni-tuebingen.de/software/megan6	A GUI, cross-platform software for taxonomic and functional analysis of metagenomic data. Supports many types of visualizations with metadata, including scatter plot, word clouds, Voronoi tree maps, clustering, and networks	(Huson et al., 2016)
MEGAHIT	https://github.com/voutcn/megahit	Ultra-fast and memory-efficient metagenomic assembler	(Li et al., 2015)
metaSPAdes	http://cab.spbu.ru/software/spades	High-quality metagenomic assembler but time-consuming and large memory requirement	(Nurk et al., 2017)
MetaQUAST	http://quast.sourceforge.net/metaquast	Evaluates the quality of metagenomic assemblies, including N50 and misassemble, and outputs PDF and interactive HTML reports	(Mikheenko et al., 2016)
MetaGeneMark	http://exon.gatech.edu/GeneMark/	Gene prediction in bacteria, archaea, metagenome and metatranscriptome. Support Linux/MacOSX system. Provides webserver for online analysis	(Zhu et al., 2010)
Prokka	http://www.vicbioinformatics.com/software.prokka.shtml	Provides rapid prokaryotic genome annotation, calls metaProdigal (Hyatt et al., 2012) for metagenomic gene prediction. Outputs nucleotide sequences, protein sequences, and annotation files of genes	(Seemann, 2014)
CD-HIT	http://weizhongli-lab.org/cd-hit	Used to construct non-redundant gene catalogs	(Fu et al., 2012)
Salmon	https://combine-lab.github.io/salmon	Provides ultra-fast quantification of reads counts of genes using a *k*-mer-based method	(Patro et al., 2017)
metaWRAP	https://github.com/bxlab/metaWRAP	Binning pipeline includes 140 tools and supports conda install, default binning by MetaBAT, MaxBin, and CONCOCT. Provides refinement, quantification, taxonomic classification and visualization of bins	(Uritskiy et al., 2018)
DAS Tool	https://github.com/cmks/DAS_Tool	Binning pipeline that integrates five binning software packages and performs refinement	(Sieber et al., 2018)

Assembly-based methods assemble clean reads into contigs using tools such as MEGAHIT or metaSPAdes (Fig. 2B). MEGAHIT is used to assemble large, complex metagenome datasets quickly using little computer memory (Li et al., 2015), while metaSPAdes can generate longer contigs but requires more computational resources (Nurk et al., 2017). Genes present in assembled contigs are then identified using metaGeneMark (Zhu et al., 2010) or Prokka (Seemann, 2014). Redundant genes from separately assembled contigs must be removed using tools such as CD-HIT (Fu et al., 2012). Finally, a gene abundance table can be generated using alignment-based tools such as Bowtie 2 or alignment-free methods such as Salmon (Patro et al., 2017). Millions of genes are normally present in a metagenomic dataset. These genes must be combined into functional annotations, such as KEGG Orthology (KO), modules and pathways, representing a form of dimensional reduction (Kanehisa et al., 2016).

In addition, metagenomic data can be used to mine gene clusters or to assemble draft microbe genomes. The antiSMASH database is used to identify, annotate, and visualize gene clusters involved in secondary metabolite biosynthesis (Blin et al., 2018). Binning is a method that can be used to recover partial or complete bacterial genomes in metagenomic data. Available binning tools include CONCOCT (Alneberg et al., 2014), MaxBin 2 (Wu et al., 2015), and MetaBAT2 (Kang et al., 2015). Binning tools cluster contigs into different bins (draft genomes) based on tetra-nucleotide frequency and contig abundance. Reassembly is performed to obtain better bins. We recommend using a binning pipeline such as MetaWRAP (Uritskiy et al., 2018) or DAStool (Sieber et al., 2018), which integrate several binning software packages to obtain refined binning results and more complete genomes with less contamination. These pipelines also supply useful scripts for evaluation and visualization. For a more comprehensive review on metagenomic experiments and analysis, we recommend Quince et al. (2017).

## Statistical analysis and visualization

**Figure 3:**
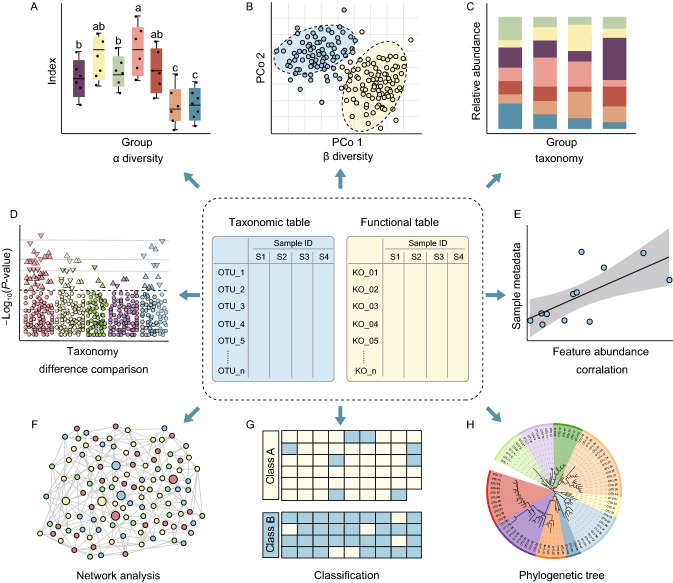
**Overview of statistical and visualization methods for feature tables.** Downstream analysis of microbiome feature tables, including alpha/beta-diversity (A/B), taxonomic composition (C), difference comparison (D), correlation analysis (E), network analysis (F), classification of machine learning (G), and phylogenetic tree (H). Please see Table 2 for more details

The most important output files from amplicon and metagenomic analysis pipeline are taxonomic and functional tables (Figs. 2 and 3). The scientific questions that researchers could answer using the techniques include the following: Which microbes are present in the microbiota? Do different experimental groups show significant differences in alpha- and beta-diversity? Which species, genes, or functional pathways are biomarkers of each group? To answer these questions, methods are needed for both overall and details statistical analysis and visualization. Overall visualization can be used to explore differences in alpha/beta- diversity and taxonomic composition in a feature table. Details analysis could involve identifying biomarkers via comparison, correlation analysis, network analysis, and machine learning (Fig. 3). We will discuss these methods below and provide examples and references to facilitate such studies (Fig. 3 and Table 2).

**Table 2:** Introduction to various analysis and visualization methods

Method	Scientific question	Visualization	Description and example reference
Alpha diversity	Within-sample diversity	Boxplot	Distribution (Edwards et al., 2015) or significant difference (Zhang et al., 2019) of alpha diversity among groups (Fig. 3A)
		Rarefaction curve	Sample diversity changes with sequencing depth or evaluation of sequencing saturation (Beckers et al., 2017)
		Venn diagram	Common or unique taxa (Ren et al., 2019)
Beta diversity	Distance among samples or groups	Unconstrained PCoA scatter plot	Major differences of samples showing group differences (Fig. 3B) or gradient changes with time (Zhang et al., 2018b)
		Constrained PCoA scatter plot	Major differences among groups (Zgadzaj et al., 2016; Huang et al., 2019)
		Dendrogram	Hierarchical clustering of samples (Chen et al., 2019)
Taxonomic composition	Relative abundance of features	Stacked bar plot	Taxonomic composition of each sample (Beckers et al., 2017) or group (Jin et al., 2017) (Fig. 3C)
		Flow or alluvial diagram	Relative abundance (RA) of taxonomic changes among seasons (Smits et al., 2017) or time-series (Zhang et al., 2018b)
		Sanky diagram	A variety of Venn diagrams showing changes in RA and common or unique features among groups (Smits et al., 2017)
Difference comparison	Significantly different biomarkers between groups	Volcano plot	A variety of scatter plots showing *P*-value, RA, fold change, and number of differences (Shi et al., 2019a)
		Manhattan plot	A variety of scatter plots showing *P*-values, taxonomy, and highlighting significantly different biomarkers (Zgadzaj et al., 2016) (Fig. 3D)
		Extend bar plot	Bar plot of RA combined with difference and confidence intervals (Parks et al., 2014)
Correlation analysis	Correlation between features and sample metadata	Scatter plot with linear fitting	Shows changes in features with time (Metcalf et al., 2016) or relationships with other numeric metadata (Fig. 3E)
		Corrplot	Correlation coefficient or distance triangular matrix visualized by color and/or shape (Zhang et al., 2018b)
		Heatmap	RA of features that change with time (Subramanian et al., 2014)
Network analysis	Global view correlation of features	Colored based on taxonomy or modules	Finding correlation patterns of features based on taxonomy (Fig. 3F) and/or modules (Jiao et al., 2016)
		Colors highlight important features	Highlighting important features and showing their positions and connections (Wang et al., 2018b)
Machine learning	Classification groups or regression analysis for numeric metadata prediction	Heatmap	Colored block showing classification results (Fig. 3G) (Wilck et al., 2017) or feature patterns in a time series (Subramanian et al., 2014).
		Bar plot	Feature importance, RA (Zhang et al., 2019), and increase in mean squared error (Subramanian et al., 2014).
Treemap	Phylogenetic tree or taxonomy hierarchy	Phylogenetic tree or cladogram	Phylogenetic tree (Fig. 3H) shows relationship of OTUs or species (Levy et al., 2018). Taxonomic cladogram highlighting interesting biomarkers (Segata et al., 2011).
		Circular tree map	Shows features in a hierarchy color bubble (Carrión et al., 2019)

Alpha diversity evaluates the diversity within a sample, including richness and evenness measurements. Several software packages can be used to calculate alpha diversity, including QIIME, the R package vegan (Oksanen et al., 2007), and USEARCH. The alpha diversity values of samples in each group could be visually compared using boxplots (Fig. 3A). The differences in alpha diversity among or between groups could be statistically evaluated using Analysis of Variance (ANOVA), Mann-Whitney U test, or Kruskal-Wallis test. It is important to note that *P*-values should be adjusted if each group is compared more than twice. Other visualization methods for alpha diversity indices are described in Table 2.

Beta diversity evaluates differences in the microbiome among samples and is normally combined with dimensional reduction methods such as principal coordinate analysis (PCoA), non-metric multidimensional scaling (NMDS), or constrained principal coordinate analysis (CPCoA) to obtain visual representations. These analyses can be implemented in the R vegan package and visualized in scatter plots (Fig. 3B and Table 2). The statistical differences between these beta-diversity indices can be computed using permutational multivariate analysis of variance (PERMANOVA) with the *adonis()* function in vegan (Oksanen et al., 2007).

Taxonomic composition describes the microbiota that are present in a microbial community, which is often visualized using a stacked bar plot (Fig. 3C and Table 2). For simplicity, the microbiota is often shown at the phylum or genus level in the plot.

Difference comparison is used to identify features (such as species, genes, or pathways) with significantly different abundances between groups using Welch’s t-test, Mann-Whitney U test, Kruskal-Wallis test, or tools such as ALDEx2, edgeR (Robinson et al., 2010), STAMP (Parks et al., 2014), or LEfSe (Segata et al., 2011). The results of difference comparison can be visualized using a volcano plot, Manhattan plot (Fig. 3D), or extended error bar plot (Table 3). It is important to note that this type of analysis is prone to produce false positives due to increases in the relative abundance of some features and decreases in other features. Several methods have been developed to obtain taxonomic absolute abundance in samples, such as the integration of HTS and flow cytometric enumeration (Vandeputte et al., 2017), and the integration of HTS with spike-in plasmid and quantitative PCR (Tkacz et al., 2018; Guo et al., 2020; Wang et al., 2020b).

**Table 3:** Useful websites or tools for reproducible analysis

Resource	Links	Description
GSA	http://gsa.big.ac.cn	HTS data deposition and sharing. Fast data transfer, interfaces in both Chinese and English, automated submission, technical support via email or QQ group, and widely recognized by international journals
Qiita	https://qiita.ucsd.edu	Platform for amplicon data deposition, analysis, and cross-study comparisons
MGnify	https://www.ebi.ac.uk/metagenomics	Webserver for amplicon and metagenomic data deposition, sharing, analysis, and cross-study comparisons
gcMeta	https://gcmeta.wdcm.org	Webserver for amplicon and metagenomic data analysis, deposition, and sharing
R Markdown	https://rmarkdown.rstudio.com	Uses a productive notebook interface to weave together narrative text and code to produce an elegantly formatted report in HTML or PDF format. Is becoming increasingly popular in microbiome research
R Graph Gallery	https://www.r-graph-gallery.com	R code for 42 chart types
GitHub	https://github.com	Online code-saving and sharing platforms with version control systems. Supports searching

Correlation analysis is used to reveal the associations between taxa and sample metadata (Fig. 3E). For example, it is used to identify associations between taxa and environmental factors, such as pH, longitude and latitude, and clinical indices, or to identify key environmental factors that affect microbiota and dynamic taxa in a time series (Edwards et al., 2018).

Network analysis explores the co-occurrence of features from a holistic perspective (Fig. 3F). The properties of a correlation network might represent potential interactions between co-occurring taxa or functional pathways. Correlation coefficients and significant *P*-values could be computed using the *cor.test()* function in R or more robust tools that are suitable for compositional data such as the SparCC (sparse correlations for compositional data) package (Kurtz et al., 2015). Networks could also be visualized and analyzed using R library igraph (Csardi and Nepusz, 2006), Cytoscape (Saito et al., 2012), or Gephi (Bastian et al., 2009). There are several good examples of network analysis, such as studies exploring the distribution of phylum or modules (Fan et al., 2019) or showing trends at different time points (Wang et al., 2019).

Machine learning is a branch of artificial intelligence that learns from data, identifies patterns, and makes decisions (Fig. 3G). In microbiome research, machine learning is used for taxonomic classification, beta-diversity analysis, binning, and compositional analysis of particular features. Commonly used machine learning methods include random forest (Vangay et al., 2019; Qian et al., 2020), Adaboost (Wilck et al., 2017), and deep learning (Galkin et al., 2018) to classify groups by selecting biomarkers or regression analysis to show experimental condition-dependent changes in biomarker abundance (Table 2).

Treemap is widely used for phylogenetic tree construction and for taxonomic annotation and visualization of the microbiome (Fig. 3H). Representative amplicon sequences are readily used for phylogenetic analysis. We recommend using IQ-TREE (Nguyen et al., 2014) to quickly build high-confidence phylogenetic trees using big data and online visualization using iTOL (Letunic and Bork, 2019). Annotation files of tree can easily be generated using the R script table2itol (https://github.com/mgoeker/table2itol). In addition, we recommend using GraPhlAn (Asnicar et al., 2015) to visualize the phylogenetic tree or hierarchical taxonomy in an attractive cladogram.

In addition, researchers may be interested in examining microbial origin to address issues such as the origin of gut microbiota and river pollution, as well as for forensic testing. FEAST (Shenhav et al., 2019) and SourceTracker (Knights et al., 2011) were designed to unravel the origins of microbial communities. If researchers would like to focus on the regulatory relationship between genetic information from the host and microorganisms (Wang et al., 2018a), genome-wide association analysis (GWAS) might be a good choice (Wang et al., 2016).

## Reproducible analysis

Reproducible analysis requires that researchers submit their data and code along with their publications instead of merely describing their methods. Reproducibility is critical for microbiome analysis because it is impossible to reproduce results without raw data, detailed sample metadata, and analysis codes. If the readers can run the codes, they will better understand what has been done in the analyses. We recommend that researchers share their sequencing data, metadata, analysis codes, and detailed statistical reports using the following steps:

### Upload and share raw data and metadata in a data center

Amplicon or metagenomic sequencing generates a large volume of raw data. Normally, raw data must be uploaded to data centers such as NCBI, EBI, and DDBJ during publication. In recent years, several repositories have also been established in China to provide data storage and sharing services. For example, the Genome Sequence Archive (GSA) established by the Beijing Institute of Genomics Chinese Academy of Sciences (Wang et al., 2017; Members, 2019) has a lot of advantages (Table 3). We recommend that researchers upload raw data to one of these repositories, which not only provides backup but also meets the requirements for publication. Several journals such as *Microbiome* require that the raw data should be deposited in repositories before submitting the manuscript.

### Share pipeline scripts with other researchers

Pipeline scripts could help reviewers or readers evaluate the reproducibility of experimental results. We provide sample pipeline scripts for amplicon and metagenome analyses at https://github.com/YongxinLiu/Liu2020ProteinCell. The running environment and software version used in analysis should also be provided to help ensure reproducibility. If Conda is used to deploy software, the command “*conda env export environment_name* > *environment.yaml*” can generate a file containing both the software used and various versions for reproducible usage. For users who are not familiar with command lines, webservers such as Qiita (Gonzalez et al., 2018), MGnify (Mitchell et al., 2020), and gcMeta (Shi et al., 2019b) could be used to perform analysis. However, webservers are less flexible than the command line mode because they provide fewer adjustable steps and parameters.

### Provide a detailed statistical and visualization reports

The tools used for statistical analysis and visualization of a feature table include Excel, GraphPad, and Sigma plot, but these are commercial software tools, and are difficult to quickly reproduce the results. We recommend using tools such as R Markdown or Python Notebooks to trace all analysis codes and parameters and storing them in a version control management system such as GitHub (Table 3). These tools are free, open-source, cross-platform, and easy-to-use. We recommend that researchers record all scripts and results of statistical analysis and visualization in R markdown files. An R markdown document is a fully reproducible report that includes codes, tables, and figures in HTML/PDF format. This work mode would greatly improve the efficiency of microbiome analysis and make the analysis process transparent and easier to understand. R visualization codes can refer to R Graph Gallery (Table 3). The input files (feature tables + metadata), analysis notebook (*.Rmd), and output results (figures, tables, and HTML reports) of the analysis can be uploaded to GitHub, which would allow peers to repeat your analyses or reuse your analysis codes. ImageGP (http://www.ehbio.com/ImageGP) provides more than 20 statistical and visualization methods, making it a good choice for researchers without a background in R.

## Notes and perspectives

It is worth noting that experimental operations have a far greater impact on the results of a study than the pipeline chosen for analysis (Sinha et al., 2017). It is better to record detailed experimental processes as metadata, which includes sampling method, time, location, operators, DNA extraction kit, batch, primers, and barcodes. The metadata can be used for downstream analyses and help researchers to determine whether these operational differences contribute to false-positive results (Costea et al., 2017). Some specific experimental steps could be used to provide a unique perspective on microbiome analysis. For example, the development and use of methods to remove the host DNA can effectively increase the proportion of the microbiome in plant endophytes (Carrión et al., 2019) and human respiratory infection samples (Charalampous et al., 2019). A large amount of relic DNA in soil can be physically removed with propidium monoazide (Carini et al., 2016). In addition, when using samples with low microbial biomass, researchers must be particularly careful to avoid false-positive results due to contamination (de Goffau et al., 2019). For these situations, DNA-free water should be used as a negative control. In human microbiome studies, the major differences in microbiome composition among individuals are due to factors such as diet, lifestyle, and drug use, such that the heritability is less than 2% (Rothschild et al., 2018). For recommendations about information that should be collected, please refer to minimum information about a marker gene sequence (MIMARKS) and minimum information about metagenome sequence (Field et al., 2008; Yilmaz et al., 2011), minimum information about a single amplified genome (MISAG) and a metagenome-assembled genome (MIMAG) of bacteria and archaea (Bowers et al., 2017), and minimum information about an uncultivated virus genome (Roux et al., 2019). In the early stage of microbiome research, data-driven studies provide basic components and conceptual frame of microbiome, however, with the development of experimental tools, more hypothesis-driven studies are needed to dissect the causality of microbiome and host phenotypes.

Shotgun metagenomic sequencing could provide insights into a microbial community structure at strain-level, but it is difficult to recover high-quality genome (Bishara et al., 2018). Single-cell genome sequencing shows very promising applications in microbiome research (Xu and Zhao, 2018). Based on flow cytometry and single-cell sequencing, MetaSort could recover high-quality genomes from sorted sub-metagenome (Ji et al., 2017). Recently developed third-generation sequencing techniques have been used for metagenome analysis, including Pacific Biosciences (PacBio) single molecule real time sequencing and the Oxford Nanopore Technologies sequencing platform (Bertrand et al., 2019; Stewart et al., 2019; Moss et al., 2020). With the improvement in sequencing data quality and decreasing costs, these techniques will lead to a technological revolution in the field of microbiome sequencing and bring microbiome research into a new era.

## Conclusion

In this review, we discussed methods for analyzing amplicon and metagenomic data at all stages, from the selection of sequencing methods, analysis software/pipelines, statistical analysis and visualization to the implementation of reproducible analysis. Other methods such as metatranscriptome, metaproteome, and metabolome analysis may provide a better perspective on the dynamics of the microbiome, but these methods have not been widely accepted due to their high cost and the complex experimental and analysis methods required. With the further development of these technologies in the future, a more comprehensive view of the microbiome could be obtained.

## References

[CR1] Alneberg J, Bjarnason BS, de Bruijn I, Schirmer M, Quick J, Ijaz UZ, Lahti L, Loman NJ, Andersson AF, Quince C (2014). Binning metagenomic contigs by coverage and composition. Nat Methods.

[CR2] Arumugam M, Raes J, Pelletier E, Le Paslier D, Yamada T, Mende DR, Fernandes GR, Tap J, Bruls T, Batto JM (2011). Enterotypes of the human gut microbiome. Nature.

[CR3] Asnicar F, Weingart G, Tickle TL, Huttenhower C, Segata N (2015). Compact graphical representation of phylogenetic data and metadata with GraPhlAn. PeerJ.

[CR4] Asshauer KP, Wemheuer B, Daniel R, Meinicke P (2015). Tax4Fun: predicting functional profiles from metagenomic 16S rRNA data. Bioinformatics.

[CR5] Bai Y, Müller DB, Srinivas G, Garrido-Oter R, Potthoff E, Rott M, Dombrowski N, Münch PC, Spaepen S, Remus-Emsermann M (2015). Functional overlap of the *Arabidopsis* leaf and root microbiota. Nature.

[CR6] Bastian M, Heymann S, and Jacomy M (2009). Gephi: an open source software for exploring and manipulating networks. In: Third international AAAI conference on weblogs and social media.

[CR7] Beckers B, Op De Beeck M, Weyens N, Boerjan W, Vangronsveld J (2017). Structural variability and niche differentiation in the rhizosphere and endosphere bacterial microbiome of field-grown poplar trees. Microbiome.

[CR8] Bertrand D, Shaw J, Kalathiyappan M, Ng AHQ, Kumar MS, Li C, Dvornicic M, Soldo JP, Koh JY, Tong C (2019). Hybrid metagenomic assembly enables high-resolution analysis of resistance determinants and mobile elements in human microbiomes. Nat Biotechnol.

[CR9] Bishara A, Moss EL, Kolmogorov M, Parada AE, Weng Z, Sidow A, Dekas AE, Batzoglou S, Bhatt AS (2018). High-quality genome sequences of uncultured microbes by assembly of read clouds. Nat Biotechnol.

[CR10] Blin K, Weber T, Lee SY, Medema MH, Pascal Andreu V, de los Santos ELC, Del Carratore F (2018). The antiSMASH database version 2: a comprehensive resource on secondary metabolite biosynthetic gene clusters. Nucleic Acids Res.

[CR11] Bolger AM, Lohse M, Usadel B (2014). Trimmomatic: a flexible trimmer for Illumina sequence data. Bioinformatics.

[CR12] Bolyen E, Rideout JR, Dillon MR, Bokulich NA, Abnet CC, Al-Ghalith GA, Alexander H, Alm EJ, Arumugam M, Asnicar F (2019). Reproducible, interactive, scalable and extensible microbiome data science using QIIME 2. Nat Biotechnol.

[CR13] Bowers RM, Kyrpides NC, Stepanauskas R, Harmon-Smith M, Doud D, Reddy TBK, Schulz F, Jarett J, Rivers AR, Eloe-Fadrosh EA (2017). Minimum information about a single amplified genome (MISAG) and a metagenome-assembled genome (MIMAG) of bacteria and archaea. Nat Biotechnol.

[CR14] Callahan BJ, McMurdie PJ, Rosen MJ, Han AW, Johnson AJA, Holmes SP (2016). DADA2: high-resolution sample inference from Illumina amplicon data. Nat Methods.

[CR15] Caporaso JG, Kuczynski J, Stombaugh J, Bittinger K, Bushman FD, Costello EK, Fierer N, Peña AG, Goodrich JK, Gordon JI (2010). QIIME allows analysis of high-throughput community sequencing data. Nat Methods.

[CR16] Carini P, Marsden PJ, Leff JW, Morgan EE, Strickland MS, Fierer N (2016). Relic DNA is abundant in soil and obscures estimates of soil microbial diversity. Nat Microbiol.

[CR17] Carrión VJ, Perez-Jaramillo J, Cordovez V, Tracanna V, de Hollander M, Ruiz-Buck D, Mendes LW, van Ijcken WFJ, Gomez-Exposito R, Elsayed SS (2019). Pathogen-induced activation of disease-suppressive functions in the endophytic root microbiome. Science.

[CR18] Charalampous T, Kay GL, Richardson H, Aydin A, Baldan R, Jeanes C, Rae D, Grundy S, Turner DJ, Wain J (2019). Nanopore metagenomics enables rapid clinical diagnosis of bacterial lower respiratory infection. Nat Biotechnol.

[CR19] Chen Q, Jiang T, Liu Y-X, Liu H, Zhao T, Liu Z, Gan X, Hallab A, Wang X, He J (2019). Recently duplicated sesterterpene (C25) gene clusters in *Arabidopsis thaliana* modulate root microbiota. Sci China Life Sci.

[CR20] Costea PI, Zeller G, Sunagawa S, Pelletier E, Alberti A, Levenez F, Tramontano M, Driessen M, Hercog R, Jung F-E (2017). Towards standards for human fecal sample processing in metagenomic studies. Nat Biotechnol.

[CR21] Csardi G, Nepusz T (2006). The igraph software package for complex network research. InterJ Complex Syst.

[CR22] de Goffau MC, Lager S, Sovio U, Gaccioli F, Cook E, Peacock SJ, Parkhill J, Charnock-Jones DS, Smith GCS (2019). Human placenta has no microbiome but can contain potential pathogens. Nature.

[CR23] de Muinck EJ, Trosvik P, Gilfillan GD, Hov JR, Sundaram AYM (2017). A novel ultra high-throughput 16S rRNA gene amplicon sequencing library preparation method for the Illumina HiSeq platform. Microbiome.

[CR24] Edgar RC (2010). Search and clustering orders of magnitude faster than BLAST. Bioinformatics.

[CR25] Edgar RC (2013). UPARSE: highly accurate OTU sequences from microbial amplicon reads. Nat Methods.

[CR26] Edgar RC, Flyvbjerg H (2015). Error filtering, pair assembly and error correction for next-generation sequencing reads. Bioinformatics.

[CR27] Edwards J, Johnson C, Santos-Medellín C, Lurie E, Podishetty NK, Bhatnagar S, Eisen JA, Sundaresan V (2015). Structure, variation, and assembly of the root-associated microbiomes of rice. Proc Natl Acad Sci USA.

[CR28] Edwards JA, Santos-Medellín CM, Liechty ZS, Nguyen B, Lurie E, Eason S, Phillips G, Sundaresan V (2018). Compositional shifts in root-associated bacterial and archaeal microbiota track the plant life cycle in field-grown rice. PLoS Biol.

[CR29] Fan K, Delgado-Baquerizo M, Guo X, Wang D, Wu Y, Zhu M, Yu W, Yao H, Zhu Y-g, Chu H (2019). Suppressed N fixation and diazotrophs after four decades of fertilization. Microbiome.

[CR30] Field D, Garrity G, Gray T, Morrison N, Selengut J, Sterk P, Tatusova T, Thomson N, Allen MJ, Angiuoli SV (2008). The minimum information about a genome sequence (MIGS) specification. Nat Biotechnol.

[CR31] Franzosa EA, McIver LJ, Rahnavard G, Thompson LR, Schirmer M, Weingart G, Lipson KS, Knight R, Caporaso JG, Segata N (2018). Species-level functional profiling of metagenomes and metatranscriptomes. Nat Methods.

[CR32] Fresia P, Antelo V, Salazar C, Giménez M, D’Alessandro B, Afshinnekoo E, Mason C, Gonnet GH, Iraola G (2019). Urban metagenomics uncover antibiotic resistance reservoirs in coastal beach and sewage waters. Microbiome.

[CR33] Fu L, Niu B, Zhu Z, Wu S, Li W (2012). CD-HIT: accelerated for clustering the next-generation sequencing data. Bioinformatics.

[CR34] Galkin F, Aliper A, Putin E, Kuznetsov I, Gladyshev VN, Zhavoronkov A (2018) Human microbiome aging clocks based on deep learning and tandem of permutation feature importance and accumulated local effects. bioRxiv 507780

[CR35] Gao L, Xu T, Huang G, Jiang S, Gu Y, Chen F (2018). Oral microbiomes: more and more importance in oral cavity and whole body. Protein Cell.

[CR36] Gonzalez A, Navas-Molina JA, Kosciolek T, McDonald D, Vázquez-Baeza Y, Ackermann G, DeReus J, Janssen S, Swafford AD, Orchanian SB (2018). Qiita: rapid, web-enabled microbiome meta-analysis. Nat Methods.

[CR37] Goodman AL, Kallstrom G, Faith JJ, Reyes A, Moore A, Dantas G, Gordon JI (2011). Extensive personal human gut microbiota culture collections characterized and manipulated in gnotobiotic mice. Proc Natl Acad Sci USA.

[CR38] Grüning B, Dale R, Sjödin A, Chapman BA, Rowe J, Tomkins-Tinch CH, Valieris R, Köster J, The Bioconda T (2018). Bioconda: sustainable and comprehensive software distribution for the life sciences. Nat Methods.

[CR39] Guo X, Zhang X, Qin Y, Liu Y-X, Zhang J, Zhang N, Wu K, Qu B, He Z, Wang X (2020). Host-associated quantitative abundance profiling reveals the microbial load variation of root microbiome. Plant Commun.

[CR40] Huang AC, Jiang T, Liu Y-X, Bai Y-C, Reed J, Qu B, Goossens A, Nützmann H-W, Bai Y, Osbourn A (2019). A specialized metabolic network selectively modulates *Arabidopsis* root microbiota. Science.

[CR41] Huang P, Zhang Y, Xiao K, Jiang F, Wang H, Tang D, Liu D, Liu B, Liu Y, He X (2018). The chicken gut metagenome and the modulatory effects of plant-derived benzylisoquinoline alkaloids. Microbiome.

[CR42] Huson DH, Beier S, Flade I, Górska A, El-Hadidi M, Mitra S, Ruscheweyh H-J, Tappu R (2016). MEGAN community edition—interactive exploration and analysis of large-scale microbiome sequencing data. PLoS Comput Biol.

[CR43] Hyatt D, LoCascio PF, Hauser LJ, Uberbacher EC (2012). Gene and translation initiation site prediction in metagenomic sequences. Bioinformatics.

[CR44] Ji P, Zhang Y, Wang J, Zhao F (2017). MetaSort untangles metagenome assembly by reducing microbial community complexity. Nat Commun.

[CR45] Jiang X, Li X, Yang L, Liu C, Wang Q, Chi W, Zhu H (2019). How microbes shape their communities? A microbial community model based on functional genes. Genom Proteom Bioinf.

[CR46] Jiao S, Liu Z, Lin Y, Yang J, Chen W, Wei G (2016). Bacterial communities in oil contaminated soils: biogeography and co-occurrence patterns. Soil Biol Biochem.

[CR47] Jin T, Wang Y, Huang Y, Xu J, Zhang P, Wang N, Liu X, Chu H, Liu G, Jiang H (2017). Taxonomic structure and functional association of foxtail millet root microbiome. Giga Sci.

[CR48] Kanehisa M, Goto S (2000). KEGG: Kyoto encyclopedia of genes and genomes. Nucleic Acids Res.

[CR49] Kanehisa M, Sato Y, Morishima K (2016). BlastKOALA and GhostKOALA: KEGG tools for functional characterization of genome and metagenome sequences. J Mol Biol.

[CR50] Kang DD, Froula J, Egan R, Wang Z (2015). MetaBAT, an efficient tool for accurately reconstructing single genomes from complex microbial communities. PeerJ.

[CR51] Klindworth A, Pruesse E, Schweer T, Peplies J, Quast C, Horn M, Glöckner FO (2012). Evaluation of general 16S ribosomal RNA gene PCR primers for classical and next-generation sequencing-based diversity studies. Nucleic Acids Res.

[CR52] Knight R, Vrbanac A, Taylor BC, Aksenov A, Callewaert C, Debelius J, Gonzalez A, Kosciolek T, McCall L-I, McDonald D (2018). Best practices for analysing microbiomes. Nat Rev Microbiol.

[CR53] Knights D, Kuczynski J, Charlson ES, Zaneveld J, Mozer MC, Collman RG, Bushman FD, Knight R, Kelley ST (2011). Bayesian community-wide culture-independent microbial source tracking. Nat Methods.

[CR54] Kurtz ZD, Müller CL, Miraldi ER, Littman DR, Blaser MJ, Bonneau RA (2015). Sparse and compositionally robust inference of microbial ecological networks. PLoS Comput Biol.

[CR55] Lagier J-C, Dubourg G, Million M, Cadoret F, Bilen M, Fenollar F, Levasseur A, Rolain J-M, Fournier P-E, Raoult D (2018). Culturing the human microbiota and culturomics. Nat Rev Microbiol.

[CR56] Langille MGI, Zaneveld J, Caporaso JG, McDonald D, Knights D, Reyes JA, Clemente JC, Burkepile DE, Vega Thurber RL, Knight R (2013). Predictive functional profiling of microbial communities using 16S rRNA marker gene sequences. Nat Biotechnol.

[CR57] Langmead B, Salzberg SL (2012). Fast gapped-read alignment with Bowtie 2. Nat Methods.

[CR58] Letunic I, Bork P (2019). Interactive tree of life (iTOL) v4: recent updates and new developments. Nucleic Acids Res.

[CR59] Levy A, Salas Gonzalez I, Mittelviefhaus M, Clingenpeel S, Herrera Paredes S, Miao J, Wang K, Devescovi G, Stillman K, Monteiro F (2018). Genomic features of bacterial adaptation to plants. Nat Genet.

[CR60] Li D, Liu C-M, Luo R, Sadakane K, Lam T-W (2015). MEGAHIT: an ultra-fast single-node solution for large and complex metagenomics assembly via succinct de Bruijn graph. Bioinformatics.

[CR61] Li J, Jia H, Cai X, Zhong H, Feng Q, Sunagawa S, Arumugam M, Kultima JR, Prifti E, Nielsen T (2014). An integrated catalog of reference genes in the human gut microbiome. Nat Biotechnol.

[CR62] Liu C, Zhou N, Du M-X, Sun Y-T, Wang K, Wang Y-J, Li D-H, Yu H-Y, Song Y, Bai B-B (2020). The mouse gut microbial **B**iobank expands the coverage of cultured bacteria. Nat Commun.

[CR63] Liu Y-X, Qin Y, Bai Y (2019). Reductionist synthetic community approaches in root microbiome research. Curr Opin Microbiol.

[CR64] Liu Y-X, Qin Y, Guo X, Bai Y (2019). Methods and applications for microbiome data analysis. Hereditas (Beijing).

[CR65] Louca S, Parfrey LW, Doebeli M (2016). Decoupling function and taxonomy in the global ocean microbiome. Science.

[CR66] Mahnert A, Moissl-Eichinger C, Zojer M, Bogumil D, Mizrahi I, Rattei T, Martinez JL, Berg G (2019). Man-made microbial resistances in built environments. Nat Commun.

[CR67] Marchesi JR, Ravel J (2015). The vocabulary of microbiome research: a proposal. Microbiome.

[CR68] McDonald D, Price MN, Goodrich J, Nawrocki EP, DeSantis TZ, Probst A, Andersen GL, Knight R, Hugenholtz P (2011). An improved Greengenes taxonomy with explicit ranks for ecological and evolutionary analyses of bacteria and archaea. ISME J.

[CR69] Members BDC (2019). Database resources of the BIG data center in 2019. Nucleic Acids Res.

[CR70] Metcalf JL, Xu ZZ, Weiss S, Lax S, Van Treuren W, Hyde ER, Song SJ, Amir A, Larsen P, Sangwan N (2016). Microbial community assembly and metabolic function during mammalian corpse decomposition. Science.

[CR71] Metsky HC, Siddle KJ, Gladden-Young A, Qu J, Yang DK, Brehio P, Goldfarb A, Piantadosi A, Wohl S, Carter A (2019). Capturing sequence diversity in metagenomes with comprehensive and scalable probe design. Nat Biotechnol.

[CR72] Mikheenko A, Saveliev V, Gurevich A (2016). MetaQUAST: evaluation of metagenome assemblies. Bioinformatics.

[CR73] Mitchell AL, Almeida A, Beracochea M, Boland M, Burgin J, Cochrane G, Crusoe MR, Kale V, Potter SC, Richardson LJ (2020). MGnify: the microbiome analysis resource in 2020. Nucleic Acids Res.

[CR74] Moss EL, Maghini DG, and Bhatt AS (2020) Complete, closed bacterial genomes from microbiomes using nanopore sequencing. Nat Biotechnol 10.1038/s41587-020-0422-6 PMC7283042 32042169

[CR75] Mu D-S, Liang Q-Y, Wang X-M, Lu D-C, Shi M-J, Chen G-J, Du Z-J (2018). Metatranscriptomic and comparative genomic insights into resuscitation mechanisms during enrichment culturing. Microbiome.

[CR76] Nguyen L-T, Schmidt HA, von Haeseler A, Minh BQ (2014). IQ-TREE: a fast and effective stochastic algorithm for estimating maximum-likelihood phylogenies. Mol Biol Evol.

[CR77] Ning K, Tong Y (2019). The fast track for microbiome research. Genom Proteom Bioinf.

[CR78] Nurk S, Meleshko D, Korobeynikov A, Pevzner PA (2017). metaSPAdes: a new versatile metagenomic assembler. Genome Res.

[CR79] Oksanen J, Kindt R, Legendre P, O’Hara B, Stevens MHH, Oksanen MJ, Suggests M (2007). The vegan package. Commun Ecol Pack.

[CR80] Parks DH, Tyson GW, Hugenholtz P, Beiko RG (2014). STAMP: statistical analysis of taxonomic and functional profiles. Bioinformatics.

[CR81] Pasolli E, Asnicar F, Manara S, Zolfo M, Karcher N, Armanini F, Beghini F, Manghi P, Tett A, Ghensi P (2019). Extensive unexplored human microbiome diversity revealed by over 150,000 genomes from metagenomes spanning age, geography, and lifestyle. Cell.

[CR82] Patro R, Duggal G, Love MI, Irizarry RA, Kingsford C (2017). Salmon provides fast and bias-aware quantification of transcript expression. Nat Methods.

[CR83] Pedersen HK, Forslund SK, Gudmundsdottir V, Petersen AØ, Hildebrand F, Hyötyläinen T, Nielsen T, Hansen T, Bork P, Ehrlich SD (2018). A computational framework to integrate high-throughput ‘-omics’ datasets for the identification of potential mechanistic links. Nat Protoc.

[CR84] Proctor LM, Creasy HH, Fettweis JM, Lloyd-Price J, Mahurkar A, Zhou W, Buck GA, Snyder MP, Strauss JF, Weinstock GM (2019). The integrative human microbiome project. Nature.

[CR85] Qian X, Liu Y-X, Ye X, Zheng W, Lv S, Mo M, Lin J, Wang W, Wang W, Zhang X (2020). Gut microbiota in children with juvenile idiopathic arthritis: characteristics, biomarker identification, and usefulness in clinical prediction. BMC Genom.

[CR86] Qin J, Li R, Raes J, Arumugam M, Burgdorf KS, Manichanh C, Nielsen T, Pons N, Levenez F, Yamada T (2010). A human gut microbial gene catalogue established by metagenomic sequencing. Nature.

[CR87] Quast C, Pruesse E, Yilmaz P, Gerken J, Schweer T, Yarza P, Peplies J, Glockner FO (2013). The SILVA ribosomal RNA gene database project: improved data processing and web-based tools. Nucleic Acids Res.

[CR88] Quince C, Walker AW, Simpson JT, Loman NJ, Segata N (2017). Shotgun metagenomics, from sampling to analysis. Nat Biotechnol.

[CR89] Ren Z, Li A, Jiang J, Zhou L, Yu Z, Lu H, Xie H, Chen X, Shao L, Zhang R (2019). Gut microbiome analysis as a tool towards targeted non-invasive biomarkers for early hepatocellular carcinoma. Gut.

[CR90] Robinson MD, McCarthy DJ, Smyth GK (2010). edgeR: a bioconductor package for differential expression analysis of digital gene expression data. Bioinformatics.

[CR91] Rognes T, Flouri T, Nichols B, Quince C, Mahé F (2016). VSEARCH: a versatile open source tool for metagenomics. PeerJ.

[CR92] Ross AA, Müller KM, Weese JS, Neufeld JD (2018). Comprehensive skin microbiome analysis reveals the uniqueness of human skin and evidence for phylosymbiosis within the class mammalia. Proc Natl Acad Sci USA.

[CR93] Rothschild D, Weissbrod O, Barkan E, Kurilshikov A, Korem T, Zeevi D, Costea PI, Godneva A, Kalka IN, Bar N (2018). Environment dominates over host genetics in shaping human gut microbiota. Nature.

[CR94] Roux S, Adriaenssens EM, Dutilh BE, Koonin EV, Kropinski AM, Krupovic M, Kuhn JH, Lavigne R, Brister JR, Varsani A (2019). Minimum information about an uncultivated virus genome (MIUViG). Nat Biotechnol.

[CR95] Saito R, Smoot ME, Ono K, Ruscheinski J, Wang P-L, Lotia S, Pico AR, Bader GD, Ideker T (2012). A travel guide to cytoscape plugins. Nat Methods.

[CR96] Salazar G, Paoli L, Alberti A, Huerta-Cepas J, Ruscheweyh H-J, Cuenca M, Field CM, Coelho LP, Cruaud C, Engelen S (2019). Gene expression changes and community turnover differentially shape the global ocean metatranscriptome. Cell.

[CR97] Seemann T (2014). Prokka: rapid prokaryotic genome annotation. Bioinformatics.

[CR98] Segata N, Izard J, Waldron L, Gevers D, Miropolsky L, Garrett WS, Huttenhower C (2011). Metagenomic biomarker discovery and explanation. Genome Biol.

[CR99] Shenhav L, Thompson M, Joseph TA, Briscoe L, Furman O, Bogumil D, Mizrahi I, Pe’er I, and Halperin E (2019) FEAST: fast expectation-maximization for microbial source tracking. Nat Methods 10.1038/s41592-019-0431-x PMC8535041 31182859

[CR100] Shi W, Li M, Wei G, Tian R, Li C, Wang B, Lin R, Shi C, Chi X, Zhou B (2019). The occurrence of potato common scab correlates with the community composition and function of the geocaulosphere soil microbiome. Microbiome.

[CR101] Shi W, Qi H, Sun Q, Fan G, Liu S, Wang J, Zhu B, Liu H, Zhao F, Wang X (2019). gcMeta: a global catalogue of metagenomics platform to support the archiving, standardization and analysis of microbiome data. Nucleic Acids Res.

[CR102] Sieber CMK, Probst AJ, Sharrar A, Thomas BC, Hess M, Tringe SG, Banfield JF (2018). Recovery of genomes from metagenomes via a dereplication, aggregation and scoring strategy. Nat Microbiol.

[CR103] Sinha R, Abu-Ali G, Vogtmann E, Fodor AA, Ren B, Amir A, Schwager E, Crabtree J, Ma S, Abnet CC (2017). Assessment of variation in microbial community amplicon sequencing by the microbiome quality control (MBQC) project consortium. Nat Biotechnol.

[CR104] Smits SA, Leach J, Sonnenburg ED, Gonzalez CG, Lichtman JS, Reid G, Knight R, Manjurano A, Changalucha J, Elias JE (2017). Seasonal cycling in the gut microbiome of the Hadza hunter-gatherers of Tanzania. Science.

[CR105] Stewart RD, Auffret MD, Warr A, Walker AW, Roehe R, Watson M (2019). Compendium of 4,941 rumen metagenome-assembled genomes for rumen microbiome biology and enzyme discovery. Nat Biotechnol.

[CR106] Stewart RD, Auffret MD, Warr A, Wiser AH, Press MO, Langford KW, Liachko I, Snelling TJ, Dewhurst RJ, Walker AW (2018). Assembly of 913 microbial genomes from metagenomic sequencing of the cow rumen. Nat Commun.

[CR107] Subramanian S, Huq S, Yatsunenko T, Haque R, Mahfuz M, Alam MA, Benezra A, DeStefano J, Meier MF, Muegge BD (2014). Persistent gut microbiota immaturity in malnourished Bangladeshi children. Nature.

[CR108] Tange O (2018). Gnu parallel 2018 (Lulu. com).

[CR109] Tierney BT, Yang Z, Luber JM, Beaudin M, Wibowo MC, Baek C, Mehlenbacher E, Patel CJ, Kostic AD (2019). The landscape of genetic content in the gut and oral human microbiome. Cell Host Microbe.

[CR110] Tkacz A, Hortala M, Poole PS (2018). Absolute quantitation of microbiota abundance in environmental samples. Microbiome.

[CR111] Truong DT, Franzosa EA, Tickle TL, Scholz M, Weingart G, Pasolli E, Tett A, Huttenhower C, Segata N (2015). MetaPhlAn2 for enhanced metagenomic taxonomic profiling. Nat Methods.

[CR112] Turnbaugh PJ, Ley RE, Hamady M, Fraser-Liggett CM, Knight R, Gordon JI (2007). The human microbiome project. Nature.

[CR113] Turner TR, Ramakrishnan K, Walshaw J, Heavens D, Alston M, Swarbreck D, Osbourn A, Grant A, Poole PS (2013). Comparative metatranscriptomics reveals kingdom level changes in the rhizosphere microbiome of plants. ISME J.

[CR114] Uritskiy GV, DiRuggiero J, Taylor J (2018). MetaWRAP—a flexible pipeline for genome-resolved metagenomic data analysis. Microbiome.

[CR115] Vandeputte D, Kathagen G, D’hoe K, Vieira-Silva S, Valles-Colomer M, Sabino J, Wang J, Tito RY, De Commer L, Darzi Y (2017). Quantitative microbiome profiling links gut community variation to microbial load. Nature.

[CR116] Vangay P, Hillmann BM, Knights D (2019). Microbiome Learning Repo (ML Repo): A public repository of microbiome regression and classification tasks. GigaScience.

[CR117] Wang J, Chen L, Zhao N, Xu X, Xu Y, Zhu B (2018). Of genes and microbes: solving the intricacies in host genomes. Protein Cell.

[CR118] Wang J, Jia Z, Zhang B, Peng L, and Zhao F (2019) Tracing the accumulation of in vivo human oral microbiota elucidates microbial community dynamics at the gateway to the GI tract. Gut, gutjnl-2019–318977 10.1136/gutjnl-2019-318977 PMC7306975 31227588

[CR119] Wang J, Thingholm LB, Skiecevičienė J, Rausch P, Kummen M, Hov JR, Degenhardt F, Heinsen F-A, Rühlemann MC, Szymczak S (2016). Genome-wide association analysis identifies variation in vitamin D receptor and other host factors influencing the gut microbiota. Nat Genet.

[CR120] Wang J, Zheng J, Shi W, Du N, Xu X, Zhang Y, Ji P, Zhang F, Jia Z, Wang Y (2018). Dysbiosis of maternal and neonatal microbiota associated with gestational diabetes mellitus. Gut.

[CR121] Wang W, Yang J, Zhang J, Liu Y-X, Tian C, Qu B, Gao C, Xin P, Cheng S, Zhang W (2020). An *Arabidopsis* secondary metabolite directly targets expression of the bacterial type III secretion system to inhibit bacterial virulence. Cell Host Microbe.

[CR122] Wang X, Wang M, Xie X, Guo S, Zhou Y, Zhang X, Yu N, and Wang E (2020b) An amplification-selection model for quantified rhizosphere microbiota assembly. Sci Bull 10.1016/j.scib.2020.03.005 36659026

[CR123] Wang Y, Song F, Zhu J, Zhang S, Yang Y, Chen T, Tang B, Dong L, Ding N, Zhang Q (2017). GSA: genome sequence archive*. Genom Proteom Bioinf.

[CR124] Ward T, Larson J, Meulemans J, Hillmann B, Lynch J, Sidiropoulos D, Spear JR, Caporaso G, Blekhman R, Knight R et al (2017) BugBase predicts organism-level microbiome phenotypes. bioRxiv 133462

[CR125] Wilck N, Matus MG, Kearney SM, Olesen SW, Forslund K, Bartolomaeus H, Haase S, Mähler A, Balogh A, Markó L (2017). Salt-responsive gut commensal modulates TH17 axis and disease. Nature.

[CR126] Wood DE, Lu J, and Langmead B (2019) Improved metagenomic analysis with Kraken 2. bioRxiv 762302 10.1186/s13059-019-1891-0 PMC6883579 31779668

[CR127] Wu Y-W, Simmons BA, Singer SW (2015). MaxBin 2.0: an automated binning algorithm to recover genomes from multiple metagenomic datasets. Bioinformatics.

[CR128] Xiao L, Feng Q, Liang S, Sonne SB, Xia Z, Qiu X, Li X, Long H, Zhang J, Zhang D (2015). A catalog of the mouse gut metagenome. Nat Biotechnol.

[CR129] Xu J, Zhang Y, Zhang P, Trivedi P, Riera N, Wang Y, Liu X, Fan G, Tang J, Coletta-Filho HD (2018). The structure and function of the global citrus rhizosphere microbiome. Nat Commun.

[CR130] Xu Y, Zhao F (2018). Single-cell metagenomics: challenges and applications. Protein Cell.

[CR131] Yang J, Yu J (2018). The association of diet, gut microbiota and colorectal cancer: what we eat may imply what we get. Protein Cell.

[CR132] Ye SH, Siddle KJ, Park DJ, Sabeti PC (2019). Benchmarking metagenomics tools for taxonomic classification. Cell.

[CR133] Yilmaz P, Kottmann R, Field D, Knight R, Cole JR, Amaral-Zettler L, Gilbert JA, Karsch-Mizrachi I, Johnston A, Cochrane G (2011). Minimum information about a marker gene sequence (MIMARKS) and minimum information about any (x) sequence (MIxS) specifications. Nat Biotechnol.

[CR134] Zgadzaj R, Garrido-Oter R, Jensen DB, Koprivova A, Schulze-Lefert P, Radutoiu S (2016). Root nodule symbiosis in Lotus japonicus drives the establishment of distinctive rhizosphere, root, and nodule bacterial communities. Proc Natl Acad Sci USA.

[CR135] Zhang F, Cui B, He X, Nie Y, Wu K, Fan D, Feng B, Chen D, Ren J, Deng M (2018). Microbiota transplantation: concept, methodology and strategy for its modernization. Protein Cell.

[CR136] Zhang J, Liu Y-X, Zhang N, Hu B, Jin T, Xu H, Qin Y, Yan P, Zhang X, Guo X (2019). *NRT1.1B* is associated with root microbiota composition and nitrogen use in field-grown rice. Nat Biotechnol.

[CR137] Zhang J, Zhang N, Liu Y-X, Zhang X, Hu B, Qin Y, Xu H, Wang H, Guo X, Qian J (2018). Root microbiota shift in rice correlates with resident time in the field and developmental stage. Sci China Life Sci.

[CR138] Zheng M, Zhou N, Liu S, Dang C, Liu Y-X, He S, Zhao Y, Liu W, Wang X (2019). N_2_O and NO emission from a biological aerated filter treating coking wastewater: main source and microbial community. J Clean Prod.

[CR139] Zhu W, Lomsadze A, Borodovsky M (2010). Ab initio gene identification in metagenomic sequences. Nucleic Acids Res.

[CR140] Zou Y, Xue W, Luo G, Deng Z, Qin P, Guo R, Sun H, Xia Y, Liang S, Dai Y (2019). 1,520 reference genomes from cultivated human gut bacteria enable functional microbiome analyses. Nat Biotechnol.

